# Novel Gene Acquisition on Carnivore Y Chromosomes

**DOI:** 10.1371/journal.pgen.0020043

**Published:** 2006-03-31

**Authors:** William J Murphy, Alison J Pearks Wilkerson, Terje Raudsepp, Richa Agarwala, Alejandro A Schäffer, Roscoe Stanyon, Bhanu P Chowdhary

**Affiliations:** 1 Department of Veterinary Integrative Biosciences, Texas A&M University, College Station, Texas, United States of America; 2 Information Engineering Branch/National Center for Biotechnology Information/National Library of Medicine, National Institutes of Health, Department of Health and Human Services, Bethesda, Maryland, United States of America; 3 Computational Biology Branch/National Center for Biotechnology Information/National Library of Medicine, National Institutes of Health, Department of Health and Human Services, Bethesda, Maryland, United States of America; 4 Department of Animal Biology and Genetics, University of Florence, Firenze, Italy; Massachusetts Institute of Technology, United States of America

## Abstract

Despite its importance in harboring genes critical for spermatogenesis and male-specific functions, the Y chromosome has been largely excluded as a priority in recent mammalian genome sequencing projects. Only the human and chimpanzee Y chromosomes have been well characterized at the sequence level. This is primarily due to the presumed low overall gene content and highly repetitive nature of the Y chromosome and the ensuing difficulties using a shotgun sequence approach for assembly. Here we used direct cDNA selection to isolate and evaluate the extent of novel Y chromosome gene acquisition in the genome of the domestic cat, a species from a different mammalian superorder than human, chimpanzee, and mouse (currently being sequenced). We discovered four novel Y chromosome genes that do not have functional copies in the finished human male-specific region of the Y or on other mammalian Y chromosomes explored thus far. Two genes are derived from putative autosomal progenitors, and the other two have X chromosome homologs from different evolutionary strata. All four genes were shown to be multicopy and expressed predominantly or exclusively in testes, suggesting that their duplication and specialization for testis function were selected for because they enhance spermatogenesis. Two of these genes have testis-expressed, Y-borne copies in the dog genome as well. The absence of the four newly described genes on other characterized mammalian Y chromosomes demonstrates the gene novelty on this chromosome between mammalian orders, suggesting it harbors many lineage-specific genes that may go undetected by traditional comparative genomic approaches. Specific plans to identify the male-specific genes encoded in the Y chromosome of mammals should be a priority.

## Synopsis

Y chromosomes are typically gene poor and enriched with repetitive elements, making them difficult to sequence by standard methods. Hence, the Y chromosome gene repertoire in mammalian species other than human has not been explored until very recently. Here the authors used a directed approach to isolate Y chromosome genes of the domestic cat, an evolutionary divergent species from human and mouse. They found that the feline Y chromosome harbors its own unique set of genes that are expressed specifically in the testes, presumably where they play an important role in spermatogenesis. Paralleling the discoveries seen from the full human Y chromosome sequence, the feline Y chromosome has acquired and remodeled some genes from autosomes, while other genes have a shared ancestry with the X chromosome. However, none of the four new genes are found on the Y chromosomes of human or mouse, although two are shared with the canine Y chromosome. This work highlights the Y chromosome as a source of potential gene novelty in different species and suggests that more directed efforts at characterizing this hitherto understudied chromosome will further enrich our understanding of the types of genes found there and the roles they may play in mammalian spermatogenesis.

## Introduction

The vast majority of our knowledge of the architecture and gene content of mammalian Y chromosomes is derived from analysis of a single species, human [1–4]. Although efforts are underway to complete the chimpanzee (the X-degenerate, or X-Y common, region was recently published [5]) and mouse Y chromosome sequences, there have been no attempts to systematically document the extent of Y-borne, species-specific genes in more divergent mammals. Despite the incredible insights into Y chromosome content and evolution provided by the genome sequence of the human MSY (the 23-Mb euchromatic, male-specific region on the Y) [4], many important questions remain that can only be addressed in a broader evolutionary context. For example, have additional X-Y common genes that have been lost in the primates and rodents been maintained in other mammalian lineages, and what are their functions? Further, if novel Y chromosome genes that enhance male reproductive function have emerged during the course of primate evolution, have similar processes shaped the Y chromosome of other mammalian lineages? If so, do they also show a limited expression pattern restricted to the testes? The answers for these questions could be derived by comparative sequencing and evolutionary analysis of Y chromosome genes in additional divergent mammalian species.

To date, however, all published comparative studies have focused on which human (or mouse) Y chromosome genes are present or not in other mammalian species [6–15]. While these studies, particularly those in marsupials and monotremes [16–20], have been extremely useful in highlighting broader evolutionary patterns, there still exists a fundamental gap in our understanding of the novel gene content and processes by which Y chromosome genes have been acquired or lost in other major lineages of mammals. Given the evidence that primates harbor multicopy Y-borne genes not found in other mammals [4,21,22], it is probable that novel genes may have been acquired or retained in other mammalian lineages.

We examined the Y chromosome gene content in the domestic cat, a species from Carnivora, a eutherian lineage divergent from primates and rodents. First, we used PCR from whole genome amplified (WGA) Y chromosome DNA to isolate and map additional X-degenerate genes that were undetected in previous studies [13]. Then we used direct cDNA selection [23–25] to provide a preliminary glimpse at a large portion of feline Y chromosome expressed sequences. Our preliminary survey of feline Y chromosome genes supports the hypothesis that while most mammalian species share a core set of X-degenerate genes, the Y chromosome of different lineages is also populated by novel multicopy gene families from diverse genomic locations that were duplicated and specialized for male-specific functions. These novel genes are expressed predominantly or exclusively in the adult testis and are multicopy, which suggests that the repetitive (ampliconic) nature of many Y chromosome genes is a widespread mechanism against degeneration in a nonrecombining environment [4].

## Results

### Isolation and Mapping of New Feline X-Degenerate Genes

**Figure 1:**
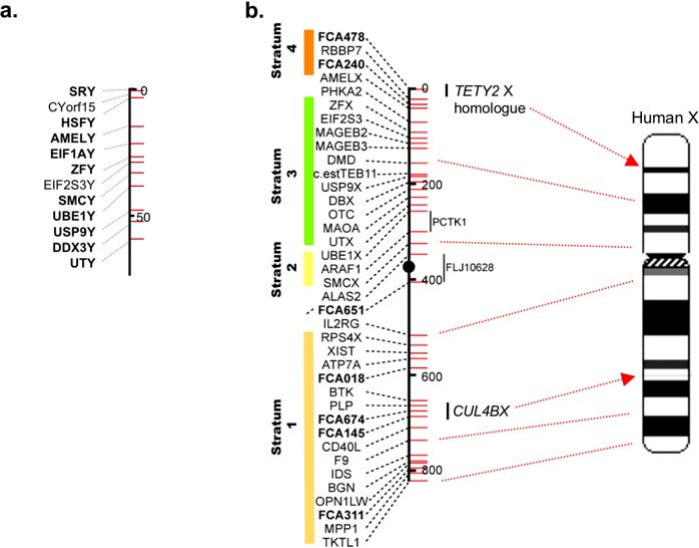
Radiation Hybrid Maps of Feline Y and X Chromosomes (A) Radiation hybrid map of single-copy, X-degenerate genes on the domestic cat Y chromosome. The scale is given in centirays (given in 50 cR_5000_ units). Markers in bold are positioned with odds of ≥1,000:1. (B) Radiation hybrid map of the domestic cat X chromosome, showing the position of X-homologous sequences of *TETY2* and *CUL4BY*. The markers are placed in their most likely intervals relative to the existing feline X chromosome RH map [13]. Red lines with arrows show the comparative position of these genes on an ideogram of the human X chromosome. Additional red dashed lines affirm the overall conservation of gene order observed between the feline and human X chromosomes [13,29,43,44]. Colored blocks on the left demarcate the relative boundaries of four of the five known human X chromosome evolutionary strata [4,26,27,51]. Note that *CUL4BX* is clearly nested in Stratum 1, consistent with its divergence from *CUL4BY* prior to the divergence of eutherians, marsupials, and monotremes approximately 230 million years ago [52] (see Table S2).

Comparative mapping studies have shown that most eutherian orders share a core set of conserved, X-degenerate, Y-borne genes [6,7,10–16]. Previous studies mapped eight of these to the feline Y chromosome [13], but efforts to isolate additional feline Y-borne loci were largely unsuccessful, primarily due to mammalian conserved X-Y primers preferentially amplifying only X-specific homologs in male cat DNA. Therefore, we used flow-sorted domestic feline Y chromosome DNA as a template for WGA, producing micrograms quantities of Y chromosome DNA. The purity and representation of the WGA Y chromosome DNA were verified by screening for the presence of the eight previously mapped Y chromosome gene sequence tagged sites (STSs). All eight STSs were successfully amplified in the WGA Y chromosome material (unpublished data). We then designed conserved primer pairs to isolate additional X-Y genes based on alignments of orthologous X- and Y-borne sequences from human, mouse, and dog genomes (Table S1). Four primer pairs were designed for the following known mammalian X-degenerate, Y chromosome genes: *EIF1AY, Eif2s3y, TMSB4Y,* and *CYorf15.* Using WGA Y chromosome DNA as a template for PCR, we amplified and sequenced each fragment and confirmed the homology of each by comparison to electronic databases (see Materials and Methods). The *TMSB4Y* primers consistently produced multiple bands and we did not pursue this gene further. Using STS primers designed from these sequences we verified that the original PCR product fragments from *EIF1AY, Eif2s3y,* and *CYorf15* gene were Y-borne, both by demonstrating male-specific amplification and by radiation hybrid (RH) mapping (Figure 1a).

### Identification of Novel Feline Y Chromosome Genes Using Direct cDNA Selection

Having identified and mapped the majority of known X-degenerate, Y-borne genes in the cat, we sought to identify the presence of ampliconic, testis-specific Y chromosome genes, using the technique of direct cDNA selection [23,25] that was successfully employed to isolate more than 90% of known human genes eventually identified in the complete MSY sequence [1,4]. Our procedure used random-primed adult normospermic feline testis cDNA and the WGA Y chromosome DNA as a selector to construct a direct selection library enriched for Y chromosome cDNAs. We sequenced 1,248 clones from this library. Exclusion of poor-quality sequences, mitochondrial DNA, autosomal contaminants, and repeat-containing sequences resulted in 580 sequences from putative Y chromosome genes (Table 1). These sequences were assembled into contigs, and STSs designed from each were used to verify their presence on the Y by male specific-PCR amplification (i.e., no product from female genomic DNA). Many of the sequences were found to be similar to previously known mammalian MSY genes by electronic database analysis, including *TSPY, CYorf15, UBE1Y,* and *HSFY*. For three of the genes *(TSPY, UBE1Y,* and *HSFY),* we were able to obtain full-length cDNA sequences and verify intact open reading frames that were conserved with their human or mouse homologs. In no cases did we observe any exons lost or duplicated between species. We were only able to obtain a partial cDNA sequence fragment for *CYorf15,* which upon comparison to the human ortholog revealed a disrupted open reading frame, raising the possibility that this cat gene product is nonfunctional.

**Table 1:**
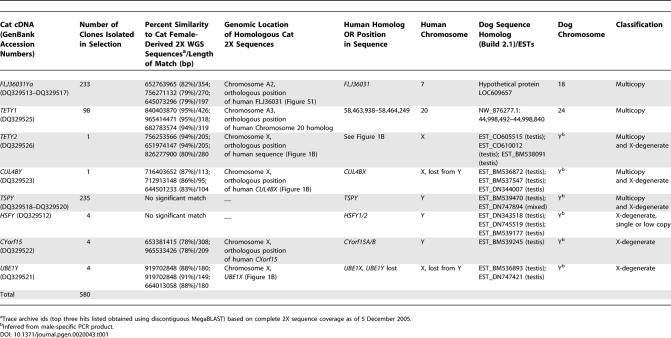
Feline Y Chromosome cDNA Clones Isolated from Direct Selection

**Figure 2:**
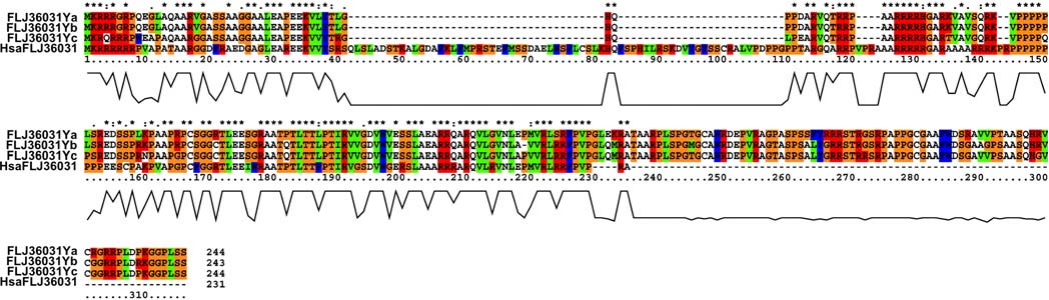
Alignment of Predicted Amino Acid Sequences for Three Feline *FLJ36031Y* Genes and Human Autosomal *FLJ36031* Above the alignments, asterisks indicate sites that are conserved. Below the alignments are histograms displaying level of amino acid conservation across the alignment. Note that the human FLJ36031 protein contains a long stretch of mostly unique amino acids from position 40 to 111, whereas the carboxyl terminus of the feline FLJ36031Y proteins (positions 232–318) is unique.

Fifty-seven percent of the feline MSY cDNA sequences isolated in the selection procedure (Table 1) derive from two genes with probable autosomal counterparts but no known X or Y-borne homolog in other eutherian mammals. The first of these is homologous to an intronless gene, *FLJ36031,* located on human chromosome 7. An autosomal version of *FLJ36031* is also found on orthologous feline and canine chromosome segments (Table 1, Figure S1). Human *FLJ36031* is single copy and expressed in several human tissues, including testes. It encodes a 231-residue protein with no detectable protein domains or motifs. The feline *FLJ36031Y* sequences were not identical but instead are a mixture of related sequences, displaying approximately 1% to 10% amino acid sequence divergence. We compared the predicted protein products of human *FLJ36031* (dog and cat autosomal sequences did not cover the complete coding sequence due to gaps) and several of the most divergent feline *FLJ36031Y* copies (Figure 2). Our alignment reveals that the feline FLJ36031Y protein has undergone considerable modification with respect to its autosomal progenitor, missing 68 interstitial residues present in the autosomal FLJ36031 proteins, while FLJ36031Y proteins have an extended carboxyl terminus that is absent from human FLJ36031. Divergence estimates based on *K*_S_ values suggest that this autosome to Y chromosome transposition event likely occurred early in eutherian evolution, prior to the carnivore radiation (Table S2).

**Figure 3:**
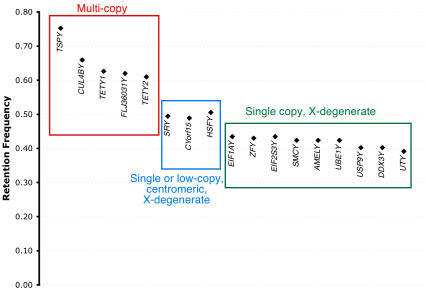
Retention Frequency of Different Classes of Cat Y Chromosome Genes in the Feline RH Panel The retention frequency of single copy X-degenerate genes (boxed in green) is similar to the genome-wide average of 0.39 [42–44]. Single copy X-degenerate genes close to the centromere (see Figure 5 for *SRY* FISH results) and potentially low–copy number genes (e.g., *HSFY*), are slightly elevated (boxed in blue). The five multicopy genes (boxed in red) have considerably higher retention frequencies than the single-copy genes, confirmed by the broad Yq chromosomal distributions determined by FISH (Figure 5).

An additional novel Y-borne transcript, termed *TETY1* (for Testis Expressed Transcript on the Y), was discovered that also had a putative autosomal origin (Table 1, Figure S2) and contains no long open reading frame (or NORF, following the usage of Skaletsky et al. [4]). A putative 120-residue protein can be predicted, although it shows no significant similarity to any known proteins (when used as query against either the protein nr database or the translated nucleotide nr database [see Materials and Methods], we found no matches with E-value less than 0.1, with a variety of parameter settings). Evolutionary comparisons to autosomal sequences support a recent autosome to Y transposition of *TETY1* during carnivore evolution, following the divergence of cat and dog lineages (Figure S2). RH mapping of male-specific STS markers for *FLJ36031Y* and *TETY1* confirmed Y-linkage, although they shared very high retention frequencies relative to the known single copy genes (Figure 3), a pattern consistent with a multicopy status [3].

### Identification of Novel X-Degenerate, Y Chromosome Genes

**Figure 4:**
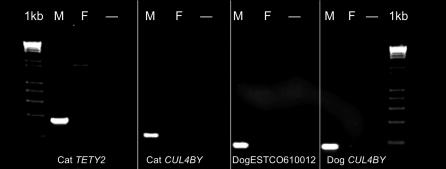
Male-Specific Amplification of *TETY2* and *CUL4BY* STS Primers in Cat and Dog A fragment of both genes has been PCR amplified with Y chromosome STS markers in matched cat and dog male and female genomic DNA samples. A 1-kilobase (kb) DNA ladder is shown to the left and right. PCR products are of the expected size in male DNAs, whereas no or nonspecific amplification is observed in female DNAs. The third lane in each set of primers (−) is a no-DNA control reaction. Dog ESTCO610012 is orthologous to cat *TETY2.*

Two Y-borne cDNA clones, *CUL4BY* and *TETY2,* showed moderate levels of sequence similarity to X chromosome sequences: (1) *CUL4BY* to *CUL4B,* a ubiquitously expressed gene on human Xq23 (henceforth named *CUL4BX*), and (2) *TETY2* to a stretch of DNA sequence situated on human Xp22.3 and lying between *APXL* and *KIAA1280* in the canine and human X chromosome assemblies (Table 1, Figures 1b and S3). Using the cat *CUL4BY* cDNA fragment to search the feline 2X whole genome sequence trace archives identified several related, but not identical, sequences that were highly similar to human and dog *CUL4BX* genes. Primers that were designed from the *CUL4BY* cDNA fragment confirmed male-specific amplification (Figure 4). We used RT-PCR and RACE (rapid amplification of cDNA ends) to obtain nearly full-length cDNA sequences of feline *CUL4BX* and *CUL4BY* that span the entire open reading frames and share 72% amino acid identity. RH mapping positioned *CUL4BX* on the long arm of the feline X chromosome, in the orthologous position of the colinear canine and human X chromosomes (Figure 1b). The physical position of *CUL4BX,* coupled with *K*_S_-based divergence estimates (Table S2), places this gene in the oldest evolutionary stratum of the mammalian X chromosome (stratum 1 of Lahn and Page [26,27]) (Figure 1b). RH mapping confirmed Y linkage for *CUL4BY,* but we could not reliably place this gene relative to other single-copy genes because of its high retention frequency, and thus probable multicopy status.

While there is no evidence for a *CUL4BY* gene or pseudogene in the complete human MSY chromosome sequence [4,28], electronic analysis of canine and feline *CUL4BX* and *CUL4BY* sequences did identify a putative genomic “fossil” located between *SRY* and *RPS4Y1* in the human and chimpanzee Y chromosome (Figure S4). This short (approximately 123 base pairs) fragment of detected homology corresponds to most of exon 12, and the following intron, of the dog *CUL4BX* gene sequence. If the hominoid Y chromosome segment containing *SRY* and *RPS4Y* are both remnants of an original chromosome block shared with the X chromosome [26], then our finding of a *CUL4BY*-like sequence in between these genes is not unexpected since *CUL4BX* was likely located between *RPS4X* and *SOX3* in the ancestral mammalian X chromosome, as it currently is on the feline, canine, and human X chromosomes [29]. We found no evidence of *CUL4BY* in the current (albeit incomplete) NCBI mouse Y chromosome sequence assembly (Build 35.1), and all mouse and rat ESTs identified by electronic database searches were identical to X-borne *Cul4b* genes. However, we did identify several canine testis ESTs that were similar to cat *CUL4BY* (Table 1) but only approximately 85% identical to canine *CUL4BX* at the nucleotide level. PCR analysis of STS primers designed from these sequences confirmed that both the cat and dog orthologous transcripts were from the Y chromosome (Figure 4).

Feline *TETY2* shows 80% sequence identity to a region of the canine X chromosome sequence between the *APXL* and *KIAA018* genes. We also identified through BLAST analysis several canine testis ESTs that were similar to feline *TETY2* but shared only 93% identity to the canine X chromosome sequence. PCR analysis of STS primers designed from these sequences confirmed that both the cat and dog orthologous transcripts were from the Y chromosome (Figure 4). RH mapping also confirmed the Y chromosome linkage for feline *TETY2,* but we were unable to reliably position the gene due to its high retention frequency (Figure 3) and multicopy status. Together, cat *TETY2* and the related dog ESTs provide evidence for a novel X-degenerate transcript, with the X counterpart either not annotated or supported by current expressed sequence tag (EST)/cDNA sequencing in dog, or possibly lost altogether. Though the homologous region of the canine X chromosome is present in the human genome, it contains only a few ESTs and no orthologous genes or transcripts precisely matching the region of X-Y homology observed in dog and cat (Figure S3). However, a putative 224-residue protein product inferred from feline *TETY2* cDNA shows similarity to a family of mouse testis-specific transcripts (hypothetical locus *LOC434881* and relatives) that span nearly two megabasepairs (Mbp) of the mouse X chromosome (approximately 147.6 Mbp to 149.5 Mbp, Build 35.1), and the cDNA matches an orthologous location on the rat X chromosome just upstream of *KIAA1280*. Evolutionary comparisons suggest that *TETY2* may have originated near the time of the carnivore radiation (Table S2) and is likely assigned to X chromosome Stratum 4 (Figure 1) [4,26].

**Figure 5:**
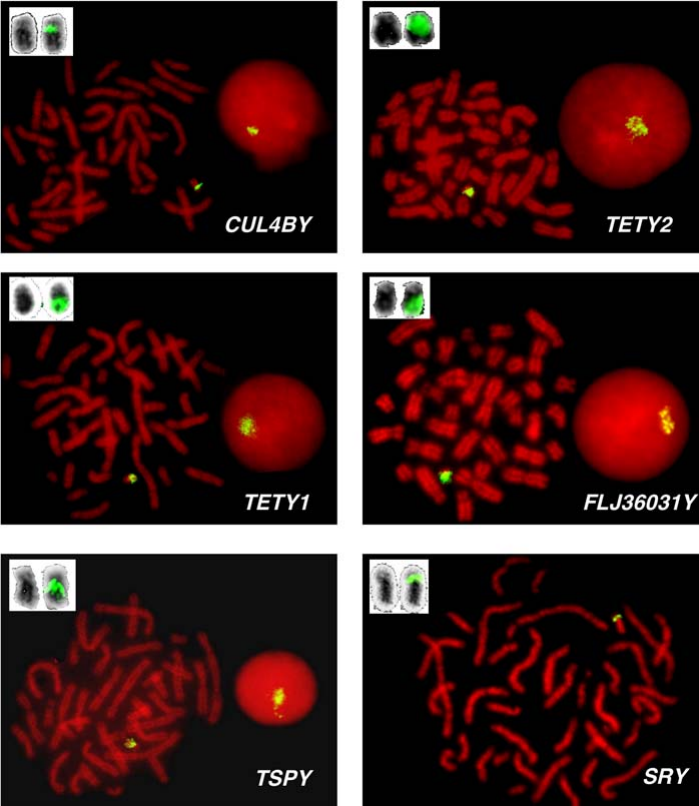
Multicopy Distribution of Novel Y Chromosome cDNAs Confirmed by FISH FISH of feline cDNA probes on male domestic cat metaphase preparations. For each novel gene, the signal is detected only on the Y chromosome and is generally restricted to the long arm. Note that no hybridization signals were found on the distal short arm, which is heterochromatic [30]. For multicopy genes we also show the hybridization results on interphase nuclei. An *SRY*-containing BAC clone hybridized to the short arm of the Y chromosome near the centromere. A magnified view of the reverse-DAPI banded Y chromosome is shown in the upper left corner of each image, both with (right) and without (left) hybridization signals.

### Fluorescent In Situ Hybridization Mapping

We used fluorescent in situ hybridization (FISH) to detect the physical location and distribution of the novel domestic feline Y chromosome genes identified in the direct cDNA selection procedure. Using cDNA clones as probes (verified as being devoid of repeats using RepeatMasker) we were able to confirm the Y chromosome origin and multicopy status of each of the four novel genes *(FLJ36031Y, CUL4BY, TETY1,* and *TETY2),* and *TSPY,* in metaphase and interphase nuclei (Figure 5). The distribution of each multicopy gene varies, from a pericentromeric pattern with *CUL4BY* to very broad signals for *TETY1, TETY2, FLJ36031Y,* and *TSPY* that cover most of the long arm of the Y chromosome. No hybridizations were found on the distal short arm of the feline chromosome, consistent with its heterochromatic composition [30]. Each gene that was determined to be multicopy based on FISH was corroborated by the presence of a high retention frequency in the cat RH panel when compared to known single copy genes (Figure 3). Using an *SRY*-containing BAC clone as a probe, we were able to demonstrate that *SRY* (the distal marker in the RH linkage group) is located on the Y chromosome short arm near the centromere (Figure 5) with a signal typical of a single copy gene. Thus, while we infer that most of the single copy X-degenerate genes are likely on the short arm, the multicopy genes are largely distributed throughout the euchromatic long arm. Based on the number of distinct full length cDNA sequences obtained from our selection experiment, we estimate that there are at least five different transcripts that encode distinct FLJ36031Y proteins and at least three different transcripts that encode distinct TSPY-like proteins. Furthermore, the broad hybridization pattern produced by cDNA probes from both *FLJ36031* and *TSPY* genes (Figure 5), indicates that each gene is present as multiple functional and/or nonfunctional copies throughout the long arm of the feline Y chromosome.

### Gene Expression

**Figure 6:**
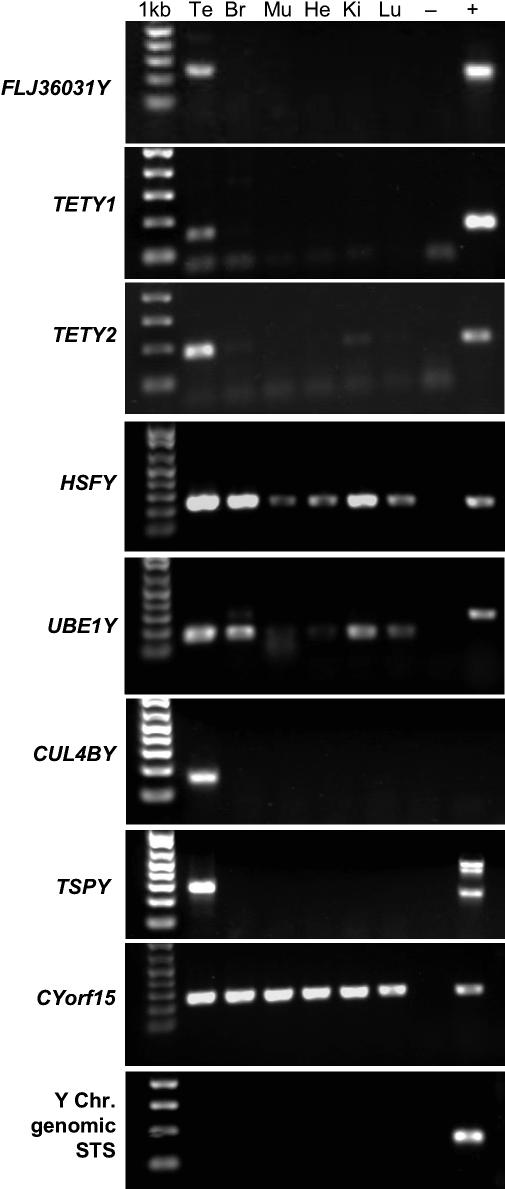
Testis-Specific Transcription of Feline Multicopy Y Chromosome Genes Amplification results of PCR primers specific for nine feline genes/sequences are shown in a panel of six adult domestic cat mRNA samples: Te, testis; Br, brain (cerebrum); Mu, muscle; He, heart; Ki, kidney; Lu, lung; −, no mRNA control; +, male domestic cat genomic DNA control. Each reaction contains 10 ng of mRNA. The gene being assayed is listed to the left of each frame. No background genomic DNA amplification was detected using a noncoding Y chromosome genomic STS marker. Negative RT reactions were also run for each STS marker and showed no amplification (unpublished data). All feline multicopy genes show testis-only expression, with the exception of *TETY2,* which also showed weak expression in kidney.

Having shown that the four new Y chromosome genes were multicopy, we sought to determine whether they also had a testis-specific gene expression profile. This prediction is based on the fact that all broadly amplified, multicopy human Y-borne genes are expressed largely or exclusively in testes [1,2,4]. Therefore we performed RT-PCR on a panel of cat mRNAs derived from six adult male domestic cat tissues. RT-PCR confirmed that all multicopy genes were expressed exclusively in testes, with the exception of *TETY2*, which showed evidence of weak expression in kidney (Figure 6). For the other X-degenerate genes obtained, we were able to demonstrate that three were broadly expressed *(CYorf15, HSFY,* and *UBE1Y),* while *TSPY* was expressed exclusively in testis (Figure 6). It remains to be determined whether the remaining feline X-degenerate genes are expressed and contain intact open reading frames.

## Discussion

Comprehensive analyses of the human Y chromosome gene content and the recent achievement of a complete chromosome sequence of the MSY have revealed four categories of mammalian Y chromosome genes [1,2,4]: (1) pseudoautosomal loci, which obligatorily recombine with X chromosome homologs and share a diverse expression and functional profile, (2) X-degenerate loci, which share a diverged X chromosome copy and are largely housekeeping genes with broad expression profiles, or in some cases have acquired more specific functions, such as *SRY,* which regulates male sex determination, (3) X-transposed loci, which have recently moved from the X to the Y, and (4) Y-specific ampliconic loci, which are expressed exclusively in testes, presumably enhance male spermatogenesis, and have been acquired from many genomic sources. The boundaries of these four categories are somewhat blurred by the finding that multicopy *RBMY, VCY,* and *TSPY* have X homologs [2,31], while multicopy *Ssty* in the mouse has a multicopy X homolog [32,33].

The ampliconic Y-borne human genes are intriguing because many have no discernible Y-borne orthologs in other eutherian mammals (except *TSPY, RBMY,* and now *HSFY*) [1,2,4]. They have been acquired from multiple locations: the *DAZ* family via transposition from an autosomal locus [22]; the *CDY* gene family through retroposition of an autosomal processed mRNA [21], and at least three X-degenerate genes, *HSFY, RBMY,* and *TSPY,* that later evolved testis-limited expression and were amplified [4,31]. The majority of ampliconic genes are members of noncoding transcription units and gene families [4]. Thus, available evidence suggests primate Y chromosomes have acquired and/or retained a diverse and unique repertoire of coding and noncoding multicopy testis-specific genes that enhance male reproductive function.

Our initial survey of the domestic feline Y chromosome transcriptome, although not comprehensive, suggests that the acquisition of genes from diverse genomic locations, followed by amplification and testis-specific expression, is characteristic of Y chromosome evolution in nonprimate eutherian mammals. It is likely that some single copy or low-copy-number feline Y chromosome genes escaped detection in our current screen. By comparison to Lahn and Page's selection search for human Y chromosome genes, we observed fewer cDNA clones from known X-degenerate genes, and no known genes from the pseudoautosomal region (PAR). These differences could be due to a number of factors, including lower sequencing depth performed here (approximately 1,200 clones versus 3,600 by Lahn and Page [1]), the possibility that some X-degenerate genes are untranscribed pseudogenes, or more pronounced differences in number between multicopy and single-copy genes in cat compared to human. Furthermore, while we detected no genes that are definitively in the human PAR, it is impossible to know whether all pseudoautosomal loci escaped detection until the PAR of the feline X chromosome is more precisely delineated by further mapping efforts.

In addition to mapping five new Y chromosome genes that were previously known in eutherians *(CYorf15, EIF1AY, Eif2s3y, HSFY,* and *TSPY),* we identified four novel, multicopy genes: *FLJ36031Y, CUL4BY, TETY1,* and *TETY2*. The multicopy status of each of these four genes, and *TSPY,* is supported by high RH panel retention frequencies relative to single copy genes (as observed in [3] and [7]), FISH results using cDNA probes, and for at least some genes (i.e., *FLJ36031* and *TSPY*), multiple divergent sequences isolated in the cDNA selection screen. It has been suggested that this multicopy feature serves as a common mechanism to maintain Y chromosome genes in a nonrecombining environment and may arise due to a number of mechanisms, including sexual antagonism, genomic conflict, and hemizygous exposure [33–35].

In our feline Y chromosome cDNA collection we observed several cases of convergent similarity to the human MSY transcriptome. For example, *CUL4BX* encodes a member of the cullin family of proteins, and is a component of the E3 ligase complex that is involved in cell cycle regulation of DNA replication through ubiquination and proteasomal degradation of target proteins [36]. Given the overall structural similarity of *CUL4BY* and *CUL4BX,* and the shared presence of a cullin domain, we infer that the *CUL4BY* encoded protein serves a related function in spermatogenesis. In humans the protein product of a unique multicopy Y chromosome gene, *BPY2*, is also involved in interaction with the E3 ligase complex [37], suggesting perhaps that Y chromosomes of different mammals were independently populated by genes with similar functional properties important for spermatogenesis.

The feline NORF transcripts identified in our survey (*TETY*1 and *TETY*2), and their broad distributions across the euchromatic pericentromeric region and the long arm of the feline Y chromosome as indicated by FISH, are reminiscent of the human MSY NORF arrays, where 13 single copy and 15 gene families encode 78 transcription units (the *TTTY* genes/gene families) that lack strong protein coding evidence [4,38]. In both cat and human these NORF transcripts are largely or exclusively expressed in testes [1,4,38]. Feline *TETY2* shows weak expression in kidney as well as testes, an expression pattern that is similar to at least two members of the human *TTTY* class of NORF genes, *TTY2L2A* and *TTY2L12A* (*sensu* [38]), and is apparently a common characteristic of some genes that have roles in sex determination [38].

Whether the broad distribution of these multicopy genes on the euchromatic long arm of the feline Y chromosome is evidence of convergently similar tandemly repeated ampliconic arrays seen in other species [4,15] must await further physical mapping and sequencing efforts. Notably, several mouse testis-specific genes *(Ssty, Sly,* and *Asty)* are each present in as many as 65 to 100+ copies on the long arm of the mouse Y chromosome [32,33]. This broad multicopy distribution has been confirmed by preliminary mouse Y chromosome sequencing data, which show that 95% of the mouse Y contains large euchromatic repeat arrays containing testis transcripts spanning the long arm [39]. We speculate that the overall mouse and feline Y chromosome architectures, which are both largely euchromatic [30,39], may be more similar to each other than either is to the human and other mammalian Y chromosomes, which contain larger proportions of heterochromatin [4,15].

We have also demonstrated that Y chromosome gene surveys in other mammalian orders will reveal additional X-degenerate genes not found in the human-mouse superorder and confirm a broader pattern of differential Y gene acquisition and/or loss across eutherian orders. For example, *RPS4Y* is only found in primates, while *AMELY* is Y-borne in many orders but absent in mouse and pig [2]. Conversely, *Eif2s3y* and *Ube1y* are Y-borne in mouse, cat, pig, and other eutherians but absent in human and some related primate lineages [2,6,12]. Our analysis of the feline Y chromosome revealed novel X-degenerate genes, including *CUL4BY,* whose counterpart, *CUL4BX,* is positioned in X chromosome Stratum 1 [26,27], and likely originated early in mammalian evolution and subsequently was lost in the human and probably rodent lineages. The older eutherian ancestry of *CUL4BY* is also supported by data mapping this gene to ampliconic contigs on the horse Y chromosome (T. Raudsepp, unpublished data). By contrast, *TETY1* and *TETY2* appear to have emerged around or after the divergence time of carnivores. These findings suggest that comparative analysis of Y chromosomes in other mammalian species may reveal many lineage- or species-specific Y chromosome genes that will likely escape detection using traditional comparative mapping approaches. The cDNA selection procedure used by Lahn and Page [1] and adapted here thus represents an efficient method to identify the unique components of the Y chromosome transcriptome in mammalian species.

## Materials and Methods

### Direct selection.

cDNA selection was performed following the protocol of Del Mastro and Lovett [25]. We synthesized cDNA from adult cat testis mRNA using random primers and Superscript II reverse transcriptase (Invitrogen, Carlsbad, California, United States), which was then adapter-ligated for PCR. After a prehybridization step with domestic cat Cot-1 DNA to block repetitive elements, the cDNA was hybridized for 40 h to biotin-labeled, WGA, Y chromosome DNA from a male domestic cat cell line (of mixed breed origin and not related to the female cat used for the cat 2X genome sequence) that was purified by fluorescence-activated cell sorting using previously described protocols [40]. Briefly, chromosome suspensions were sorted using a dual-laser sorter (FACS Vantage SE; Becton-Dickinson [BD], Palo Alto, California, United States) into 30 μl of water (approximately 10,000 of each chromosome). WGA reactions were performed with the GenomiPhi amplification system (Amersham Biosciences, Little Chalfont, United Kingdom) and 2 μl of the chromosome suspension. PCR-amplified primary selected cDNA was purified and then subjected to a second round of selection. The final secondary selection PCR-amplified cDNAs were cloned en masse into the TOPO-TA cloning vector (Invitrogen). Then 1,248 plasmid clones were picked and grown overnight at 37 °C in 2-ml, 96-well culture plates containing LB media plus ampicillin (50 μg/ml). Plasmid DNA was isolated with an alkaline lysis–based kit (REAL-prep96; Qiagen, Valencia, California, United States), directly sequenced with universal primers and Big Dye-Terminator (Applied Biosystems, Foster City, California, United States) sequencing chemistry, and resolved on an ABI-3730 capillary sequencing apparatus. After trimming vector and poor quality sequence, and removal of repeat-containing sequences (identified with RepeatMasker at http://www.RepeatMasker.org, using the cat repeat library but retaining simple repeats and low complexity sequence), the remaining cDNA sequences were assembled into contigs using the Sequencher software program (GeneCodes, Ann Arbor, Michigan, United States). We attempted to obtain full-length sequences for each novel Y-borne transcript using RACE.

### RH mapping.

For each novel Y gene, a Y-specific STS primer pair was designed in Primer 3 [41] and physically mapped using the domestic cat RH panel [42]. X-degenerate genomic survey sequences derived from introns *(EIF1AY, EIF2S3Y,* and *CYorf15)* were masked for feline repetitive elements prior to primer design. All primers were tested for Y-specificity by amplifying male and female DNAs in parallel. PCR-based genotyping was performed on an expanded set of 178 clones from the feline 5,000-rad whole genome RH panels in 10-μl reaction volumes with standardized conditions [43,44].

Markers were ordered using a reduction from the RH mapping problem to the traveling salesman problem as encoded in rh_tsp_map [45] and using similar methods to those in [44]. We used the three variants of the maximum likelihood criterion, all of which agreed on the data in this study. The instances were solved to optimality using CONCORDE [46] linked to the QSopt library (http://www2.isye.gatech.edu/~wcook/qsopt) for integer programming. The initial step set aside markers that are too close to another marker and saved these for placement later. The remaining, eligible markers were ordered in an “MLE-consensus” map, which means that all three formulations of the MLE criterion give the same optimal solution. Markers set aside initially were then placed in an interval of the MLE-consensus map by the placement program in rh_tsp_map. Finally, the placed markers were assigned a preferred centiray position using the RHMAXLIK program in the RHMAP software package [47].

### RNA extraction, cDNA synthesis, and RT-PCR.

mRNA from six flash-frozen, adult male domestic shorthair cat tissue samples (testes, brain, muscle, heart, kidney, and lung) was extracted using Invitrogen's FastTrack 2.0 Kit. RT-PCR was performed using Invitrogen's Superscript III One-Step RT-PCR System with Platinum *Taq*. Reactions were performed in a final volume of 15 μl at the manufacturer's specifications, with 40 pmol of each primer and 10 ng of DNase-treated mRNA. PCRs using mRNA as a template and Y chromosome STS primers derived from extragenic DNA revealed no background DNA contamination in mRNA preparations (Figure 6). Reactions for genomic controls included 50 ng of DNA, 1.5 mM MgCl_2_, 40 pmol of each primer, and 0.3 unit of Platinum *Taq* in a final reaction volume of 15 μl. RT-PCR conditions started with a 30-min incubation at 50° C, followed by a 2-min hot-start incubation at 94 °C. Cycling conditions involved 35 cycles of 15 s of denaturation at 94 °C, 15 to 30 s of annealing at 55 to 60 °C, and 30 to 60 s of extension at 68 °C, followed by a final extension of 5 min at 68 °C. Genomic controls were run simultaneously with the cDNA samples. Products were visualized on 1.5% gels with ethidium bromide in 1× TBE buffer. Products were either cloned (into the Invitrogen TOPO TA vector) or purified directly using Microcon-PCR filters (Millipore) and then sequenced (as described above).

### 5′ and 3′ RACE.

Rapid amplification of cDNA ends was done using Invitrogen's 3′ RACE and GeneRacer systems at the manufacturer's specifications and 1 μg of total testis RNA. First-round PCR cycling conditions for 3′ RACE used a 2-min hot-start at 94 °C, followed by 35 cycles of 30 s of denaturation at 94 °C, 30 s of annealing at 58° C, and 3 min of extension at 72 °C. A final extension of 10 min at 72 °C completed the reaction. A nested gene-specific primer was used for a subsequent PCR performed under identical conditions but with 30 cycles. Template for 3′ RACE nested PCR was 2 μl of a 1:100 dilution of first round product. Cycling conditions for the GeneRacer reactions followed the manufacturer's suggested profiles, with the following changes: the annealing was decreased 2 °C every five cycles, for the first ten cycles, from 72 °C to 70 °C, followed by 20 final cycles with an annealing temperature of 68 °C. Nested GeneRacer reactions (using 1 μl of the first round product) used 25 cycles with an annealing temperature of 65 °C. Products were visualized on a 1% to 1.5% gel with ethidium bromide in 0.5× TBE buffer. PCR products were cloned using Invitrogen's TOPO TA Cloning Kit for Sequencing. Transformed cells were plated on LB agar with 50 μg/ml ampicillin. Colonies were picked after an overnight incubation at 37 °C and cultured overnight at 37 °C in LB medium with 50 μg/ml ampicillin. Plasmid DNA was extracted using either Invitrogen's SNAP Miniprep Kit or Qiagen's REAL Prep 96-well Kit. Plasmid DNAs were digested with EcoR1 and visualized on a 1% agarose gel with ethidium bromide in 1× TBE buffer. Plasmids containing appropriate sized inserts were sequenced using universal primers as described above.

### PCR amplification and DNA sequencing.

Conserved X-Y primers were designed using multispecies alignments and optimized using a touchdown PCR strategy. PCRs were performed with Ampli*Taq* Gold DNA polymerase (Applied Biosystems) and varying magnesium concentrations. All fragments were purified with Microcon-PCR devices (Millipore) and sequenced in both directions using BigDye terminator chemistry. Sequencing reactions were purified using SephadexG50 columns and resolved on ABI3100 or 3730 capillary sequencers.

### FISH mapping.

A large insert clone containing the feline *SRY* gene was obtained by screening the feline RPCI-86 BAC library with an *SRY* STS probe [13,48]. BAC clone DNA was isolated as described [15], and the presence of *SRY* was verified via PCR. DNA from cDNA clones (not containing repetitive DNA) were labeled with biotin-16-dUTP or digoxygenin-11-dUTP using nick translation (Roche Molecular Biochemicals). Hybridization to male domestic cat (mixed breed) metaphase chromosomes was performed as described [15]. A minimum of 20 metaphase spreads and 20 interphase spreads were captured and analyzed for each experiment with a Zeiss Axioplan2 fluorescent microscope equipped with Cytovision/Genus application software, Version 2.7 (Applied Imaging, San Jose, California, United States).

### BLAST analyses and divergence estimates.

Nucleotide BLAST searches against the domestic cat trace archives (WGS) and dog, human, mouse, and rat assemblies were undertaken using discontiguous MegaBLAST optimized for cross-species comparison [49] using an E-value of 10. Protein BLAST [50] searches were performed against the protein nr database or translated nucleotide nr database. We used the program DIVERGE in the GCG software (Genetics Computer Group) to estimate rates of synonymous substitutions per synonymous sites (*K*_S_), and nonsynonymous substitutions per nonsynonymous sites (*K*_A_), excluding gaps and correcting for multiple hits. Approximate divergence estimates between X-Y and X-autosome gene pairs were made assuming that *K*_S_ values are roughly proportional to age [4,26,27] and were calibrated using the 50– to 60–million years ago divergence between cats and dogs, applied to the *TETY1*-canine chromosome 24 gene pair (Figure S2, Table S2).

## Supporting Information

Figure S1 Genomic Mapping of Feline Autosomal *FLJ36031* to Feline Chromosome A2 (128 KB PDF)

Figure S2 Multispecies Alignment and Phylogeny of *TETY1* and Orthologous Human (Hsa), Canine (Cfa), and Feline Autosomal Genome Fragments (102 KB PDF)

Figure S3 Alignments of TETY2 Homologs to Canine and Human X Chromosomes (264 KB PDF)

Figure S4 Evidence for a *CUL4BY* Genomic Fossil on Human (left) and Chimpanzee (right) Y Chromosomes (93 KB PDF)

Table S1 Primers Used for This Study (32 KB PDF)

Table S2 Sequence Divergence between X/Y and Y-Autosome (Y/A) Gene Pairs (79 KB PDF)

### Accession Numbers

All sequences have been deposited in GenBank (http://www.ncbi.nlm.nih.gov/Genbank) under accession numbers DQ329512–DQ329529 (see Table 1).

## Abbreviations

EST: expressed sequence tag
FISH: fluorescent in situ hybridization
RACE: rapid amplification of cDNA ends
RH: radiation hybrid
STS: sequence tagged site
WGA: whole genome amplified/amplification
