# Supplementary material for: Novel Gene Acquisition on Carnivore Y Chromosomes
Source: PLoS Genet. 2006 Mar 31;2(3):e43. doi: 10.1371/journal.pgen.0020043 (PMC1420679; doi:10.1371/journal.pgen.0020043)
Supplement: Figure S1 — (128 KB PDF) [file pgen.0020043.sg001.pdf]

Fig. S1. Genomic mapping of feline autosomal *FLJ36031* to cat chromosome A2. Position of the feline autosomal *FLJ36031* STS (Table S1) in the cat A2 radiation hybrid map (A2-RH) [44], shown in red. The genetic linkage maps (A2-GL) are shown to the left, and the homologous synteny blocks (HSB) with the human genome (colored based on chromosome, which is labeled in the center of each block) are shown to the right. Physical coordinates in the human genome are given at the ends of each HSB.

a.

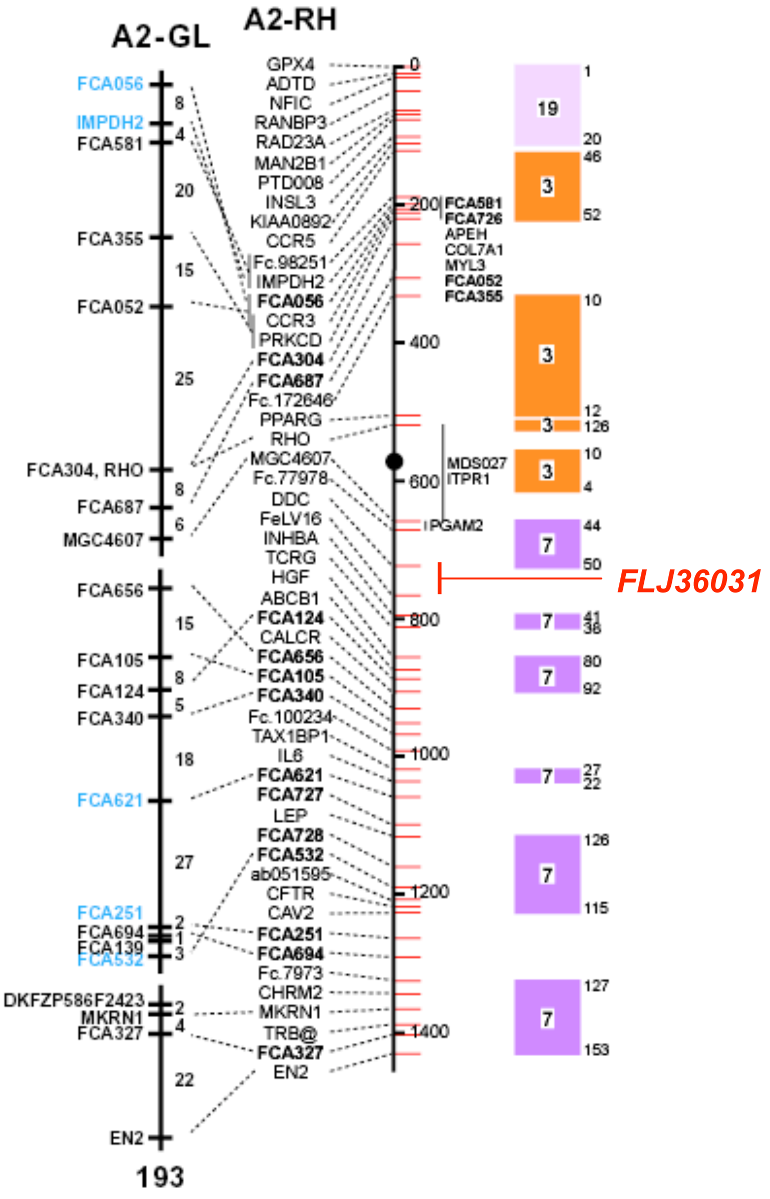

b.

```
>MegaBLAST alignment of cat FLJ36031Y to feline chromosome A2
sequence trace(id 652763965)
 Score =  310 bits (161),  Expect = 3e-81
  Identities = 295/356 (82%), Gaps = 23/356 (6%)
  Strand=Plus/Minus

 Query  670   GGGACCGCG-TCCCCTAGTGCAC-TTTATGGGCGAAGAAGCACCAGAGGGT-CCCGGCCC  726
              ||||||| | | ||||||||| | |||| ||| ||||||||||| ||   || |||||||
 Sbjct  748   GGGACCGGGTTTCCCTAGTGC-CTTTTACGGGTGAAGAAGCACCGGA-TGTCCCCGGCCG  691

 Query  727   GCGCCCCCGGGATGCGGCGCCGCCTTCTGGGACTCCGGGGCAGCTGGGCCCTCTGCTGCC  786
              | |||||| ||||||||||||||| ||||||||||||||||| || ||||||||||||
 Sbjct  690   GGGCCCCCCGGATGCGGCGCCGCCGTCTGGGACTCCGGGGCAGGTGTGCCCTCTGCTGCC  631

 Query  787   TCCCAGCACCGGGTCTGCGGAGGGAGGAGACCCCTGGACC-GCAAGGGAGGTCCCCTTTC  845
              |||||| ||||||| |||||||||||||||   ||||||| ||||||| || ||||||||
 Sbjct  630   TCCCAGTACCGGGTTTGCGGAGGGAGGAGACAGCTGGACCCGCAAGGGTGGCCCCCTTTC  571

 Query  846   TTCCTAACCCCAGATTGAGAGGTCTCCAGGACTGTAGGGGCAGATCCATGTTCTCTCTTG  905
              ||||||||||||||||||| |||||||||||| |||||||||||| | ||||| |||
 Sbjct  570   TTCCTAACCCCAGATTGAGACGTCTCCAGGACTATAGGGGCAGATCCGTTTTCTCCCTTT  511

 Query  906   T----TC--CCTCATTGCTCTG-AAGTCGTCAGAAGAAGCCATTCCACCCCCACCCCCTC  958
              |    ||  ||||| || |||| ||| | || ||||||| |||||              ||
 Sbjct  510   TGGACTCTACCTCACTGGTCTGGAAGCCCTCCGAAGAAGTCATTCTTTTT--------TC  459

 Query  959   CAAAGGAATTTGGTTCCATGCTTGTGTAATAAAGCTAGAGTTT-CTTTGCTGTTCA  1013
              ||||||||||||||| | |||||||||||||||||||| ||| ||| ||||||||
 Sbjct  458   CAAAGGAATTTGGTTTCGTGCTTGTGTAATAAAGCTAGAATTTACTT-GCTGTTCA  404
```
