# Supplementary material for: Novel Gene Acquisition on Carnivore Y Chromosomes
Source: PLoS Genet. 2006 Mar 31;2(3):e43. doi: 10.1371/journal.pgen.0020043 (PMC1420679; doi:10.1371/journal.pgen.0020043)

Fig. S2. Multispecies alignment and phylogeny of *TETY1* and orthologous human (Hsa), canine (Cfa), and feline autosomal genome fragments. a) Alignment is shown with respect to positions 327-744 of feline *TETY1*. DNA fragments correspond to positions 58,463,864-58,464,280 of human chr. 20 (Hsa20) (May 2004 Build), 47,950,680-47,951,088 on dog chr. 24 (Cfa24) (July 2004 Build), and 200-618 of cat trace archive sequence ti840403870 from cat chromosome A2. **b)** Maximum Likelihood based phylogeny of the aligned sequences demonstrates the recent phylogenetic relationship of feline *TETY1* and the feline A2 sequence.

a.

```
TETY1            TGTACCATTGTCTTCAGAAGAGA
gnl|ti|840403870 TGTACCATTGTCTTCAGAAGAGA
Hsa20            AGTGCAGTTGTGTTCAGAAGGGA
Cfa24            TGTACCATTGTCTTCAGGAGAAG

TETY1            GTCTCCTGAGAGTCCTGTGCTGTCTGAACCTCGCTGCTGTGCCCCTGTCC
gnl|ti|840403870 GTCTCCTGAGAGCCCTGTGCTGTCTGAGCCTCGCTACTGTGCCCCTGTCC
Hsa20            GTCTCATGAGGACCGGGGCCACTTGGAGCCTCAGTGCTATTTTCGTGTCA
Cfa24            GTCTCCTGAGAGCCCTGTGCTGTCTGAGCCTCGCTGCCGTTTTTCTGTTA

TETY1            ACAGACAATGGTGGGAACAAAG-TTGCCCTCATTACAGATGTGCAGGGGA
gnl|ti|840403870 ACAGACAAAGGTGGGAACAAAG-TTGCCCTCATTACAGATGTGCAGGGGA
Hsa20            GCAGACAAAAGTGGGAACAAAGCTTGCCTTCGTTTCAGATATGCAGGGGA
Cfa24            GCAGG-AAAGGTGGGAACAAAG-CCACCCTCATTACAGGTGAGCAGGGCA

TETY1            GCGCCCTGCTGTAGGGAAACTTCAGGAAACACTCGGACACTGAACAATGG
gnl|ti|840403870 GCACCCTGCTGTAGGGAAACTTCAGGAAACACTCGGAAAGTGAACAATGG
Hsa20            GCTTGCTGCTGCACG-AAGCTCTCAGAAATACTCGGAAGATGAACAATGG
Cfa24            GCACCCCACTGAATAGAAACTTCAGGAAACACTCTGTGAGTGAACAGTGG

TETY1            CTCCCRGAGAACAGGAACA-GATACGCCTCAATTTCAGAAACAAATGCAC
gnl|ti|840403870 CTCCCAGAGAACAGGAACA-GATACGCCTCAATTTCAGAAACAAATGCAC
Hsa20            CTCCCAGAGAACATGAGCAAGACACATTTCAATTTCAGAAACAAATGCAC
Cfa24            CTCCCGGAGAGCATGAGCA-TCTACGCCTCAATTTCAGAAACATATGCAC

TETY1            GTGAAAGATCATCGTTTATTGGAGGACATATATATTAACTTAAACTCTTT
gnl|ti|840403870 ATGAAAGATCATCTTTTATTGGAGAACATATATATTAACTTAAACTCTTT
Hsa20            ATGAAAGATCATCTTTTATTGGAGAACATACGT--TAAGTTATCCTCTTT
Cfa24            ATGAAAGATCATCTTTTATTGGAGAACATGTAT--TAACTTACACTCTTT

TETY1            TCAAAAA-CGTAACCAGGAAACAGTGACCTTTCCAACGCCTGCCTGAGCT
gnl|ti|840403870 TAAAAAA-CATAACCAGGAAACAGTGACCTTTCCAACGCCTTCATGAGCT
Hsa20            TCAAAAA-C--AACCAGGAAACAGTGACCTTTCCAATGCCTTCATCAGCT
Cfa24            TAAAAAAACATAACTGGGAAACAGTGACCTTTCCAATGCCTTCATGAGCC

TETY1            GCGGTTGTAGGAGAAGATAATAAATGCCATGAGACGCTCTGGAACAGAAA
gnl|ti|840403870 GCAGTTTTAGGAGAACATAATAAATGCCACGAGACGCTCTGGAACAGAAA
Hsa20            GCAGTTTTAGGAGAACATAATAAATGCTACTAGACACCGTGGAGCAGAAA
Cfa24            GCAGTTTTAGGAGAACATAATAAATGCCACTAGATACCGCGGAACAGAAA

TETY1            ACATAGGGATTCAACAAATGAA-CTCGGGA-CACACGGCAAAGATACCCA
gnl|ti|840403870 ACATAGGGATTCAACAAATGAAACTCGGGA-CACACGGCAAAGACACCCA
Hsa20            ACACAAGGATTCAACAAATTAAACTTTGGAACACACACCAAAGACACCCA
Cfa24            ACATAAAGATTCGACAAATTAAACTTTGTAACACACACCAA---------
```

b.

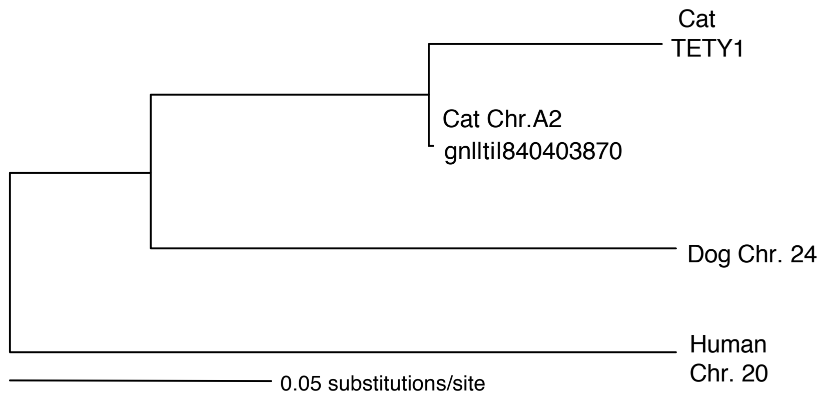

Supplement: Figure S2 — (102 KB PDF) [file pgen.0020043.sg002.pdf]
