# Supplementary material for: Novel Gene Acquisition on Carnivore Y Chromosomes
Source: PLoS Genet. 2006 Mar 31;2(3):e43. doi: 10.1371/journal.pgen.0020043 (PMC1420679; doi:10.1371/journal.pgen.0020043)

Fig. S3. Alignments of TETY2 homologs to canine and human X chromosomes

a. Conservation of cat *TETY2* cds to canine chrX. Matching bases in *TETY2* cDNA and canine genomic sequences are colored blue and capitalized. Light blue bases mark the boundaries of gaps in either sequence (often splice sites). The dog ESTs (Table 1) collectively match a larger span of the dog X chromosome (pos. 6,507,232-6,534,282) which was used in the Blat search to the human X chromosome in b (bottom of page).

(positions 6,568,881-6,569,818bp in dog July 2004 X assembly):

```
attttatgtt ttctctttgg tgttcccggc ttaacttcac gatgcctcta  6568931
ccttgtcgtt agtcaacccg aagtgaagca ctattttctc aaactgacca  6568981
GCCTcTTTTT CAGTTATtTG GCCTGGGCTc ctGGCATCAG cCTAGCtggc  6569031
AGCccATCCT GGtGCagGTG GGAGTTgAAg AGCAACATCA TTGGACTtTG  6569081
GGAAGCTcTC cAgTTTCAAA AATTgaaagc tctggctccA TTGTTGAGTT  6569131
GCAGGATCAA TGGgAaCTGG GTCATTTCAc ataaAAATCT GGTATGGGCA  6569181
GGaCCTGATA TTGcgGGCAA ATTtTaTcAA gTCaGTAGTC CTGATTTTTG  6569231
GcTCgGTGGC ACTTtTGtTC tGCaGGATCC ATGCCCCgTg CCCAGATTTC  6569281
CTTtGATcaT GTTACAACAT CTCTGCCTTC TTTCtttctt tttttttttt  6569331
ttaagatttt atttatttat tcatgagaga cacacagaga gagagaagca  6569381
gagacacagg cagagggaga agcaggctcc acgcagggaa cccgatgtgg  6569431
gacttgatcc caggactcca ggatcaggcc ctaggctgaa ggcaagcact  6569481
aaaccgctga gccacccagg gatccctctg ccttctttct tgtctTCAgC  6569531
TGTAGTTacA CTGCaATTAC AGTGAGCTGG AATTTTGCTG TGGATTTTTA  6569581
TGGCCAAACC ACCCcTGAcT TCCCACTTAG cTTTCCCAcT aGgAAAGAAA  6569631
TGCTcagaag gaaatgcttc tacatgTTCC CACTAGGAcT CACggCTGCC  6569681
ACCCTCTCGC TAATAACTGC CgccATGTTC TtCTGTGaaa tattttctgt  6569731
agaacagtgg agtcaatgaa acccaaggtt gtggccaagt gtcccaaaca  6569781
caaggtctga acgaagggcc tactctttag aatgact
```

b. *TETY2* X homologous region in the human genome. Region of X chromosome homology to the Y-linked transcript *TETY2*, displayed in the UCSC Genome Browser Viewer (May 2004 assembly). The canine X chromosome region homologous to canine and feline *TETY2* ESTs and cDNAs (Table 1 & Fig.4) was used as the input sequence for a BLAT search (versus the human X chromosome shown). Chromosome coordinates (in base-pairs) are given above.

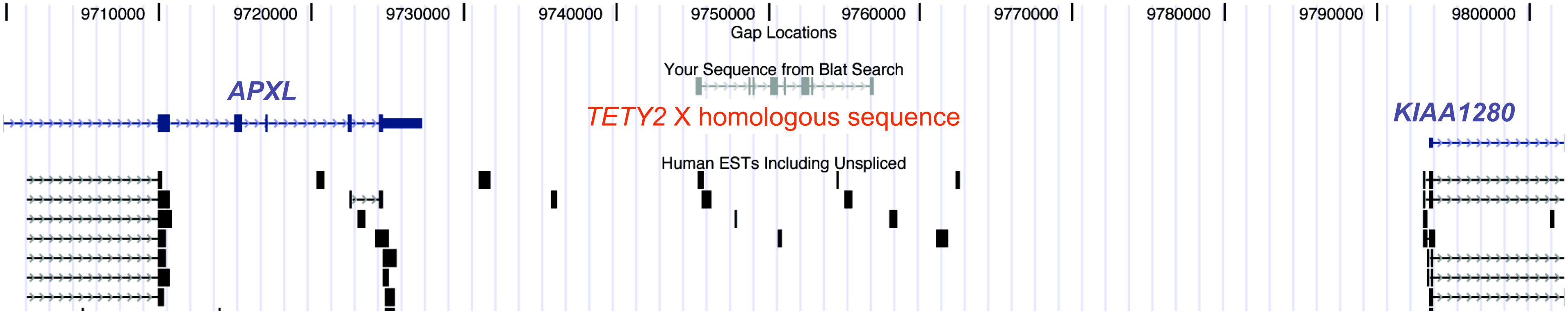

Supplement: Figure S3 — (264 KB PDF) [file pgen.0020043.sg003.pdf]
