# Supplementary material for: Novel Gene Acquisition on Carnivore Y Chromosomes
Source: PLoS Genet. 2006 Mar 31;2(3):e43. doi: 10.1371/journal.pgen.0020043 (PMC1420679; doi:10.1371/journal.pgen.0020043)
Supplement: Figure S4 — (93 KB PDF) [file pgen.0020043.sg004.pdf]

Fig. S4. Evidence for a *CUL4BY* genomic fossil on human (left) and chimp (right) Y chromosomes. The green box shows the position of a Y chromosome fragment with sequence similarity (shown below) to both the canine and feline *CUL4BY* cDNAs, and the canine X chromosome *CUL4BX* gene annotation in Build 1 of the canine genome.

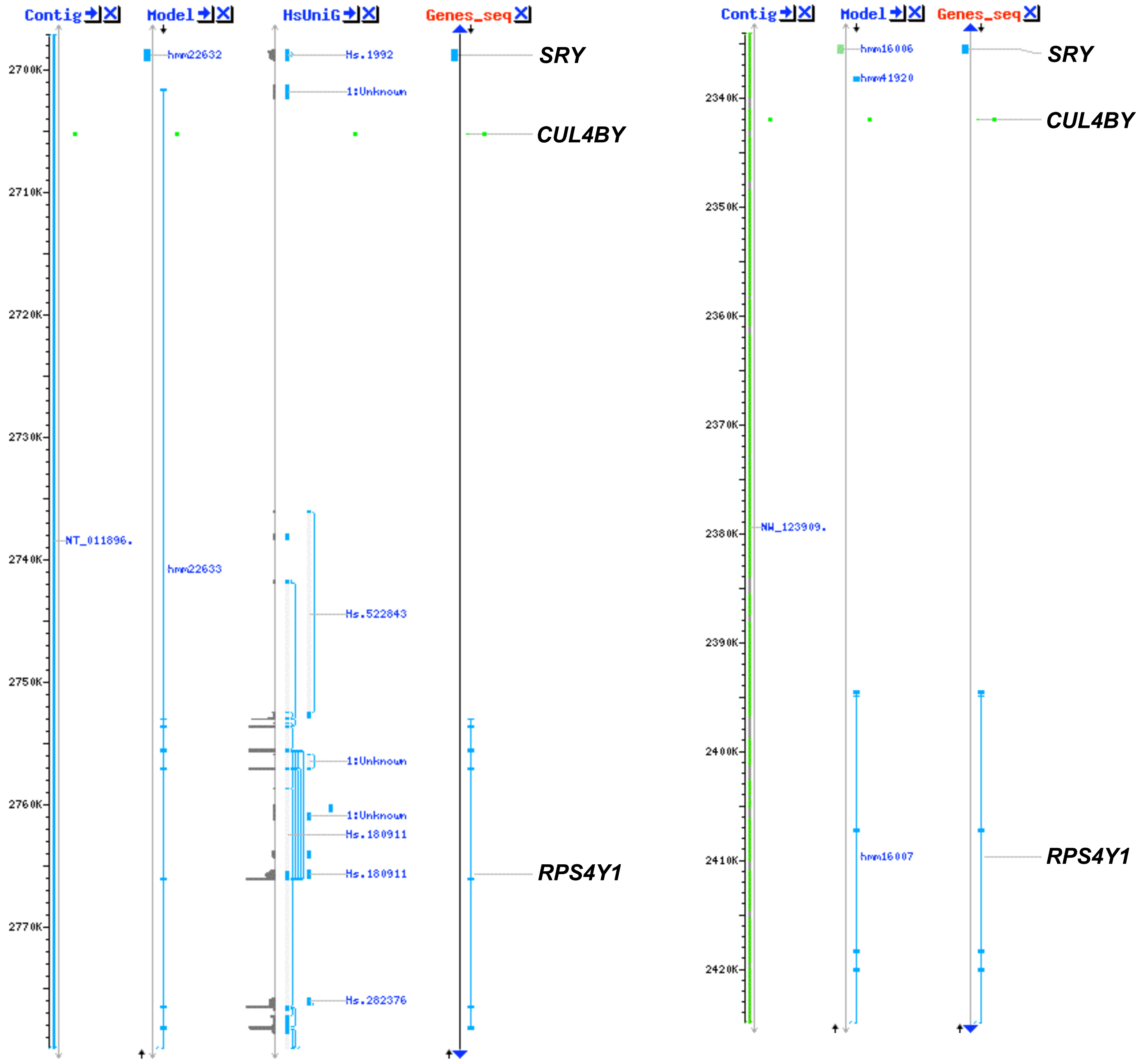

Alignment of Human.chrY and canine chrX:95574167-95574288

(Upper case nucleotides indicate sites of conservation between the dog and human sequences)

Human.chrY

```
AAaCcTtGtt CAGGtcACAA aGAAGCTACA GATGAAGAAC acGAAAAAAt TGTTGGtTAA  2705215
AATAaaActA TAacTAGgcT TATtTAcGGT gAGTAaTTTc tTTTCATgCT cCaTTTAAA
```

Dog.chrX (reverse strand):

```
AAgCtTcGcg CAGGcaACAA gGAAGCTACA GATGAAGAAC ttGAAAAAAT GTTGGaTAAA  31338869
AtTAtgAtcA TAttTAGatT TATcTAtGGT aAGTAtTTTt gTTTtaaaAT tCTtCtTTTA  31338809
AA
```

Side by Side Alignment*

```
02705156 aaaccttgttcaggtcacaaagaagctacagatgaagaacacgaaaaaattgttggttaa 02705215
<<<<<<<< || | | |  ||||  |||| |||||||||||||||||||  ||||||| |||||| ||| <<<<<<<<
31338928 aagcttcgcgcaggcaacaaggaagctacagatgaagaacttgaaaaaa-tgttggataa 31338870

02705216 aa-taaaactataactaggcttatttacggtgagtaatttcttttc---atgctccattt 02705271
<<<<<<<< || ||  |  |||  |||  |||| || ||| |||| |||  |||    || || | ||| <<<<<<<<
31338869 aattatgatcatatttagatttatctatggtaagtatttttgttttaaaattcttctttt 31338810

02705272 aaa 02705274
<<<<<<<< ||| <<<<<<<<
31338809 aaa 31338807
```
