# Supplementary material for: Novel Gene Acquisition on Carnivore Y Chromosomes
Source: PLoS Genet. 2006 Mar 31;2(3):e43. doi: 10.1371/journal.pgen.0020043 (PMC1420679; doi:10.1371/journal.pgen.0020043)
Supplement: Table S1 — (32 KB PDF) [file pgen.0020043.st001.pdf]

**Table S1. Primers used for this study**				
**Gene**	**X-Y common forward primer**	**X-Y common reverse primer**	**Male-specific/RH mapping forward primer**	**Male-specific/RH mapping reverse primer**
EIF1AY	AAARTACAATGCAGATGAAGCTAGAA	CATCATCATCTCCAGGACCA	TGAGAGGATTTTAGCAATTTTCAA	TGCCTTTAGAAAGAAATGTCTCC
EIF2S3Y	CCAGTRCCYCYAAGAGACTTTACTTCA	CRACTTCACAGCCAGGTTTG	TAAGGGTGAGAGCAGGCATT	CATCCCAATCCCTAATGCAG
CYorf15	TGATATTCTTCAACATCAAGACTCA	GAGTGTTTAGGGCTTGCATCA	TGTGGTTAGGTGAAAACACACC	TGATTCCTATGACCAGGCTTC
HSFY			TGGATCAGCATGCCATTTTA	CTGGCTGACACAGCAGGATA
TSPY			CTAGAGCCGGCAAATCTGTG	TGACAACAGCAGCCTCAACT
FLJ36031Y			GCCTGAGGAGAAGGTGTTGT	GCACCTTTCTCTGGCTGACC
FLJ36031			AGCCCTCCGAAGAAGTCATT	TCATGAGTTTCTGCCATTTCC
TETY1			CAGATGTGCAGGGGAGCAC	AATATATATGTCCTCCAATAAACGATG
TETY2			GCTTTGGGCATCAGTCTAGC	AAATGACCCAGCTTCCATTG
CUL4BY			ATAAACCCCTGCCACTGGA	TCTTCCAAGCTGTATTTAACAGAGA
CUL4BY-dog			GAAGAGGCATGGCAAAAACT	TCCAAGCTGTACTGAACAGAGG
estCO610012-dog (*TETY2* homolog)			AAGGACACCTCCTTCCTTCTTC	CTGGCTGGCATTGCAGTT
