# Supplementary material for: Novel Gene Acquisition on Carnivore Y Chromosomes
Source: PLoS Genet. 2006 Mar 31;2(3):e43. doi: 10.1371/journal.pgen.0020043 (PMC1420679; doi:10.1371/journal.pgen.0020043)
Supplement: Table S2 — (79 KB PDF) [file pgen.0020043.st002.pdf]

Table S2: Sequence divergence between X/Y and Y-autosome (Y/A) gene pairs.

Gene pair	Chr	Coding sequence compared (bp)$	Ks (s.d.)	Ka (s.d.)	Ks/Ka	Approx. Divergence Date#	% DNA divergence	Accession numbers of gene pair	Cat positions compared	Human/Dog homologous positions
SRY / Dog *SOX3*	X/Y	261	1.06 (0.33)	0.20 (0.05)	5.4	241-286	26.8	NM_001009240/NW_879563.1	148-408	61764640-61764377
CUL4BY / *CUL4BX*	X/Y	2,325	0.88 (0.09)	0.16 (0.02)	5.5	200-238	23.8	DQ329523/DQ329524	119-2449/307-2655	
TETY2 / Dog Chr. X*	X/Y	465	0.18 (0.06)	0.13 (0.03)	1.4	41-49	13.4	DQ329526/NW_879562.1	629-1114	6533434-6534110
TETY1 / Dog Chr. 24	Y/A	138	0.22 (0.12)	0.23 (0.07)	1.0	50-60	19.6	DQ329525/NW_876277.1	325-462	44998492-44998628
FLJ36031Ya / Human FLJ36031	Y/A	222	0.33 (0.13)	0.24 (0.07)	1.4	75-89	22.1	DQ329513/NM_175884	118-339	262-699
FLJ36031Ya / Dog FLJ36031*	Y/A	222	0.29 (0.13)	0.26 (0.08)	1.2	66-78	22.1	DQ329513/NW_876265.1	118-339	13746018-13746436

* Feline autosomal sequences (WGS trace archives) not available or too short for accurate comparison of putative coding sequences. See Methods for complete details.

Calibrated using the *TETY1*/Dog Chr. 24 Ks, and cat-dog divergence date range of 50-60 Mya [Springer *et al*. (2003) *Proc Natl Acad Sci USA* 100:1056-1061], see Fig. S2

$ Excluding gaps
